# Supplementary material for: Amotl1 mediates sequestration of the Hippo effector Yap1 downstream of Fat4 to restrict heart growth
Source: Nat Commun. 2017 Feb 27;8:14582. doi: 10.1038/ncomms14582 (PMC5333361; doi:10.1038/ncomms14582)
Supplement: Supplementary Information — Supplementary figures and supplementary tables. [file ncomms14582-s1.pdf]

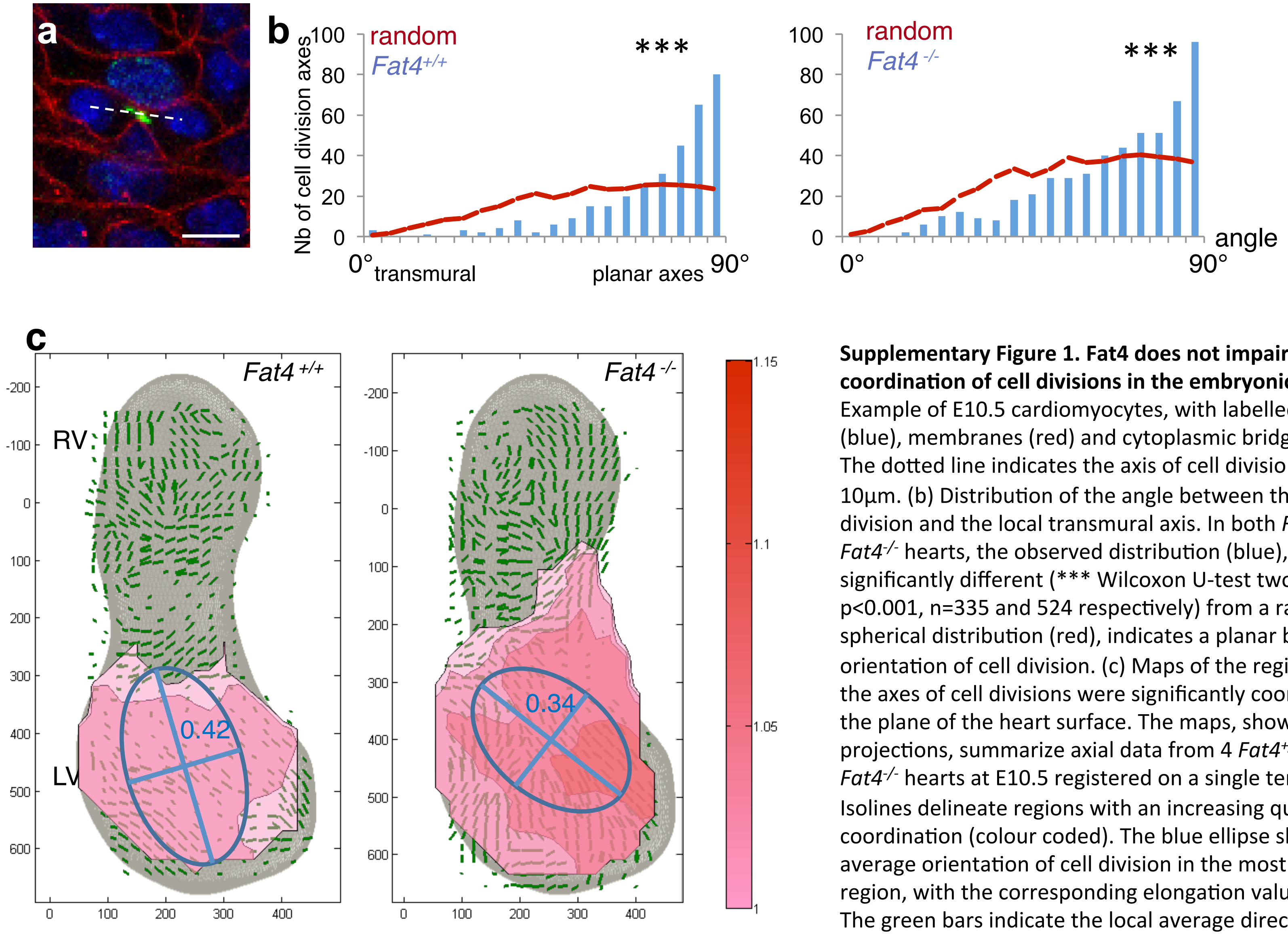


**Supplementary Figure 1. Fat4 does not impair the coordination of cell divisions in the embryonic heart.** (a) Example of E10.5 cardiomyocytes, with labelled nuclei (blue), membranes (red) and cytoplasmic bridge (green). The dotted line indicates the axis of cell division. Scale bar : 10µm. (b) Distribution of the angle between the axis of cell division and the local transmural axis. In both *Fat4*$^{+/+}$ and *Fat4*$^{-/-}$ hearts, the observed distribution (blue), which is significantly different (*** Wilcoxon U-test two-tailed, $p<0.001$, $n=335$ and 524 respectively) from a random spherical distribution (red), indicates a planar bias of the orientation of cell division. (c) Maps of the regions where the axes of cell divisions were significantly coordinated in the plane of the heart surface. The maps, shown as XY projections, summarize axial data from 4 *Fat4*$^{+/+}$ and 3 *Fat4*$^{-/-}$ hearts at E10.5 registered on a single template. Isolines delineate regions with an increasing quality of axial coordination (colour coded). The blue ellipse shows the average orientation of cell division in the most extensive region, with the corresponding elongation value $1-(E_2/E_1)$. The green bars indicate the local average direction per 100µm x 100µm box, shifted every 20 µm. Nb, number ; LV: left ventricle; RV: right ventricle.

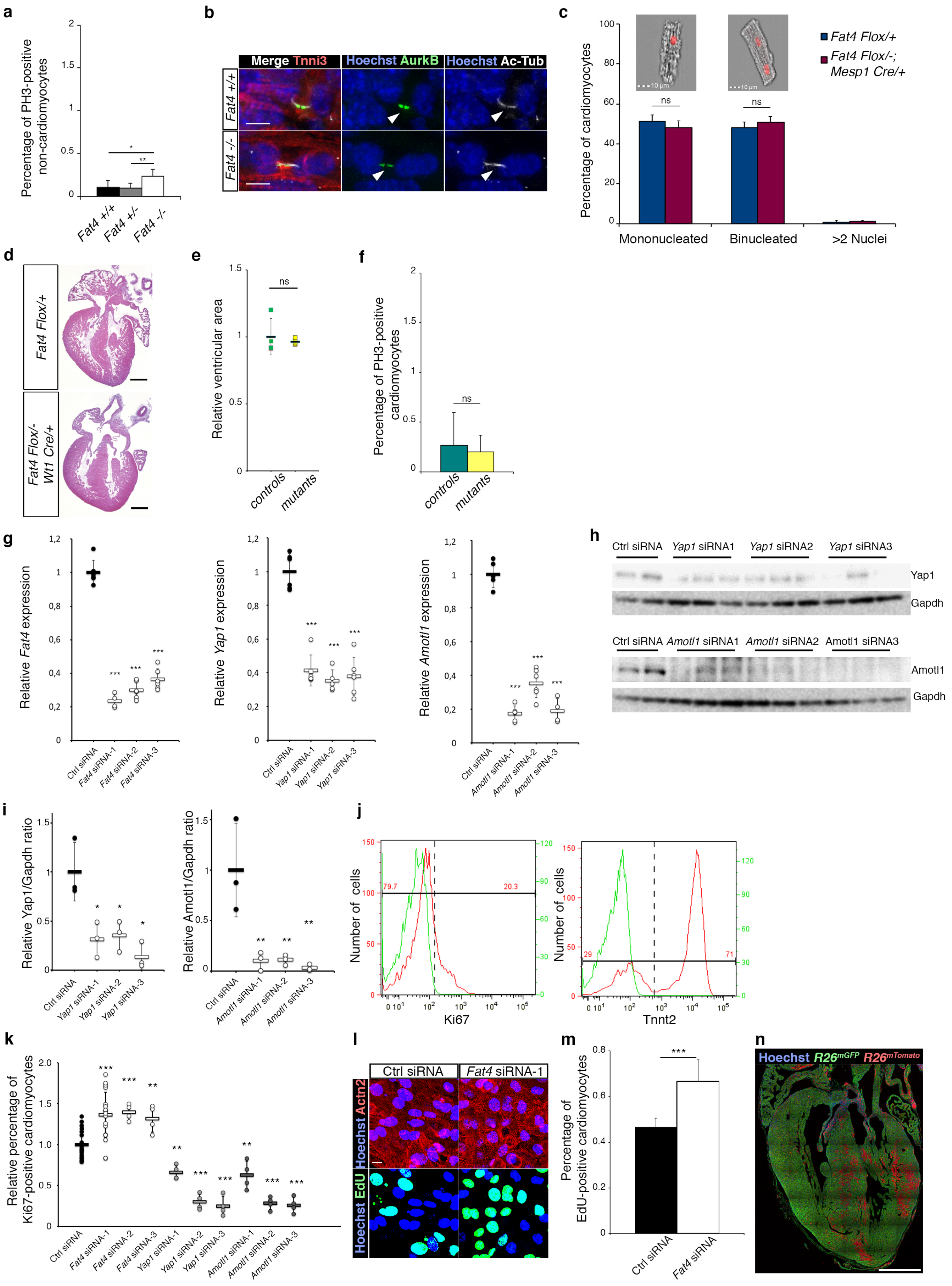

**Supplementary Figure 2. Proliferation of cardiac cells and RNA interference.** (a) Quantification of the number of mitotic non-cardiomyocytes positive for PH3 in *Fat4*$^{+/+}$ (n=6), *Fat4*$^{+/-}$ (n=6) and *Fat4*$^{-/-}$ (n=5) hearts at P0 (see Figure 2a). * p<0.05, ** p<0.01 (Anova). (b) Immunodetection in *Fat4*$^{+/+}$ and *Fat4*$^{-/-}$ hearts at P0 of cardiomyocytes, marked by cardiac troponin I (Tnni3), undergoing cytokinesis, marked by cytoplasmic bridges (arrowheads) positive for AuroraB kinase (Aurkb) and acetylated tubulin (Ac-tub). (c) Quantification of the number of nuclei per cardiomyocyte in control *Fat4*$^{Flox/+}$ (n=6) and mutant *Fat4*$^{Flox/-}$*;Mesp1*$^{Cre/+}$ (n=6) hearts at P14. (d) Histological sections of control *Fat4*$^{Flox/+}$ and mutant *Fat4*$^{Flox/-}$*;Wt1*$^{Cre/+}$ hearts at P0. (e) Corresponding quantification of the ventricular thickening (n=4 ctrl, 4 mutant hearts). (f) Quantification of the number of mitotic cardiomyocytes positive for PH3 in *Fat4*$^{Flox/+}$, (controls, n=5) and *Fat4*$^{Flox/-}$*;Wt1*$^{Cre/+}$ (mutants, n=5) hearts at P0. (g) Efficient down-regulation of *Fat4* (n= 6 ctrl, 4 *Fat4* siRNA-1, 6 siRNA-2, 6 siRNA-3 cultures), *Yap1* (n= 6 ctrl, 6 *Yap1* siRNA-1, 5 siRNA-2, 6 siRNA-3) or *Amotl1* (n= 5 ctrl, 6 *Amotl* siRNA-1, 6 siRNA-2, 4 siRNA-3) relative to *Gapdh* transcripts, in primary cultures of cardiomyocytes treated with 3 different siRNA for each gene. *** p<0.001 (Anova). (h) Western blot showing efficient down-regulation of Yap1 and Amotl1 proteins relative to Gapdh in cultures treated with the indicated siRNA. (i) Corresponding quantifications (n= 3 cultures in each condition). * p<0.05, ** p<0.01 (Anova). (j) Profile by flow cytometry of primary cell cultures stained with Ki67 and Tnnt2 (cardiac troponin T), using an isotype antibody as a negative control (green) or a specific primary antibody (red). (k) Percentage of proliferating Ki67-positive cardiomyocytes (Tnnt2-positive) after treatment with different *Fat4*, *Yap1* and *Amotl1* siRNAs (n=20 cultures for Ctrl and *Fat4* siRNA-1, n=4 in each other condition). ** p<0.01, *** p<0.001 (Anova). (l) Immunodetection of replicating EdU-positive cardiomyocytes when *Fat4* is down-regulated, with the corresponding quantification in (m) (n=6 cultures in each condition). *** p<0.001 (Student test). (n) Histological section of a *Mesp1*$^{Cre/+}$ *; R26*$^{mTmG/+}$ E18.5 heart, showing 70% of cells (n=3 hearts) in which the Cre has been active (green). Ctrl, control. Scale bars : 5 $\mu$m in (b), 500 $\mu$m in (d, n), 10$\mu$m in (c, l). ns, no significant difference (Student test).

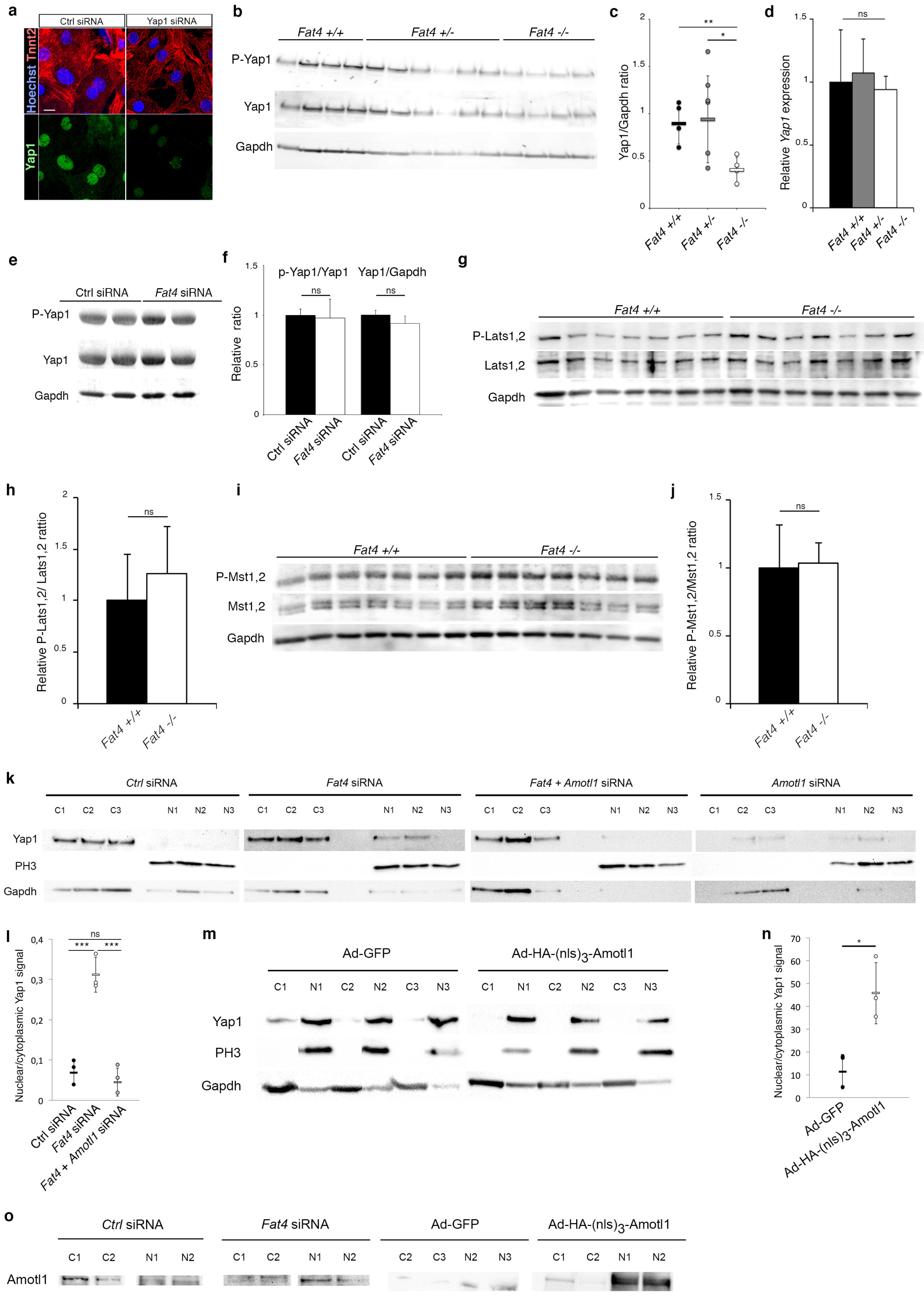


**Supplementary Figure 3. Normal canonical Hippo signalling when *Fat4* expression is impaired and nuclear translocation of Yap1 and Amotl1.** (a) Control of Yap1 immunodetection in cardiomyocytes treated with the indicated siRNA. (b) Western blot showing normal Yap1 phosphorylation at the Hippo kinase target site, in hearts at P0. See quantification in Fig. 4f. (c) The stability of Yap1 is decreased in *Fat4*$^{-/-}$ (n=4) compared to control (n=4 *Fat4*$^{+/+}$, n=6 *Fat4*$^{+/-}$) hearts, suggesting feedback mechanisms. * p<0.05, ** p<0.01 (Anova). (d) Expression of *Yap1* is not affected in *Fat4*$^{-/-}$ (n=7) compared to control (n=5 *Fat4*$^{+/+}$, n=5 *Fat4*$^{+/-}$) hearts at P0. (e) Western blot of extracts of primary cultures of cardiomyocytes treated with *Fat4* siRNA. (f) Normal Yap1 phosphorylation and Yap1 levels were quantified when *Fat4* expression is down-regulated (n=7 cultures in each condition). (g) Western blot, quantified in (h), showing no change in the phosphorylation of the Hippo kinases Lats1,2, between *Fat4*$^{+/+}$ (n=7) and *Fat4*$^{-/-}$ (n=7) hearts at P0. (i) Western blot, quantified in (j), showing no change in the phosphorylation of the Hippo kinases Mst1,2 between *Fat4*$^{+/+}$ (n=7) and *Fat4*$^{-/-}$ (n=7) hearts at P0. Bands in the same lane of Western blots were from the same blot, retreated after stripping (see Fig. S5). (k) Fractionated Western blots, quantified in (l, n=3 numbered cultures in each condition), showing increased nuclear localisation of Yap1 in primary cultures of cardiomyocytes treated with *Fat4* siRNA compared to controls or to a genetic rescue with *Amotl1* siRNA. p<0.001 (Anova). (m) Fractionated Western blot, quantified in (n, n=3 numbered cultures in each condition), showing increased nuclear localisation of Yap1 in primary cultures of cardiomyocytes infected with nuclear Amotl1 (Ad-HA-(nls)3-Amotl1) compared to controls (Ad-GFP). * p<0.05 (Student test). (o) Fractionated Western blot, showing increased nuclear localisation of Amotl1 in primary cultures of cardiomyocytes treated with *Fat4* siRNA compared to control siRNA or infected with nuclear Amotl1 (Ad-HA-(nls)3-Amotl1) compared to control infections (Ad-GFP). The same fractions (C, cytoplasmic, N, nuclear) as in (k) and (m) were used. Ctrl, control ; ns, no significant difference (Anova in d, Student test in f, h, j). Scale bar : 10 $\mu$m.

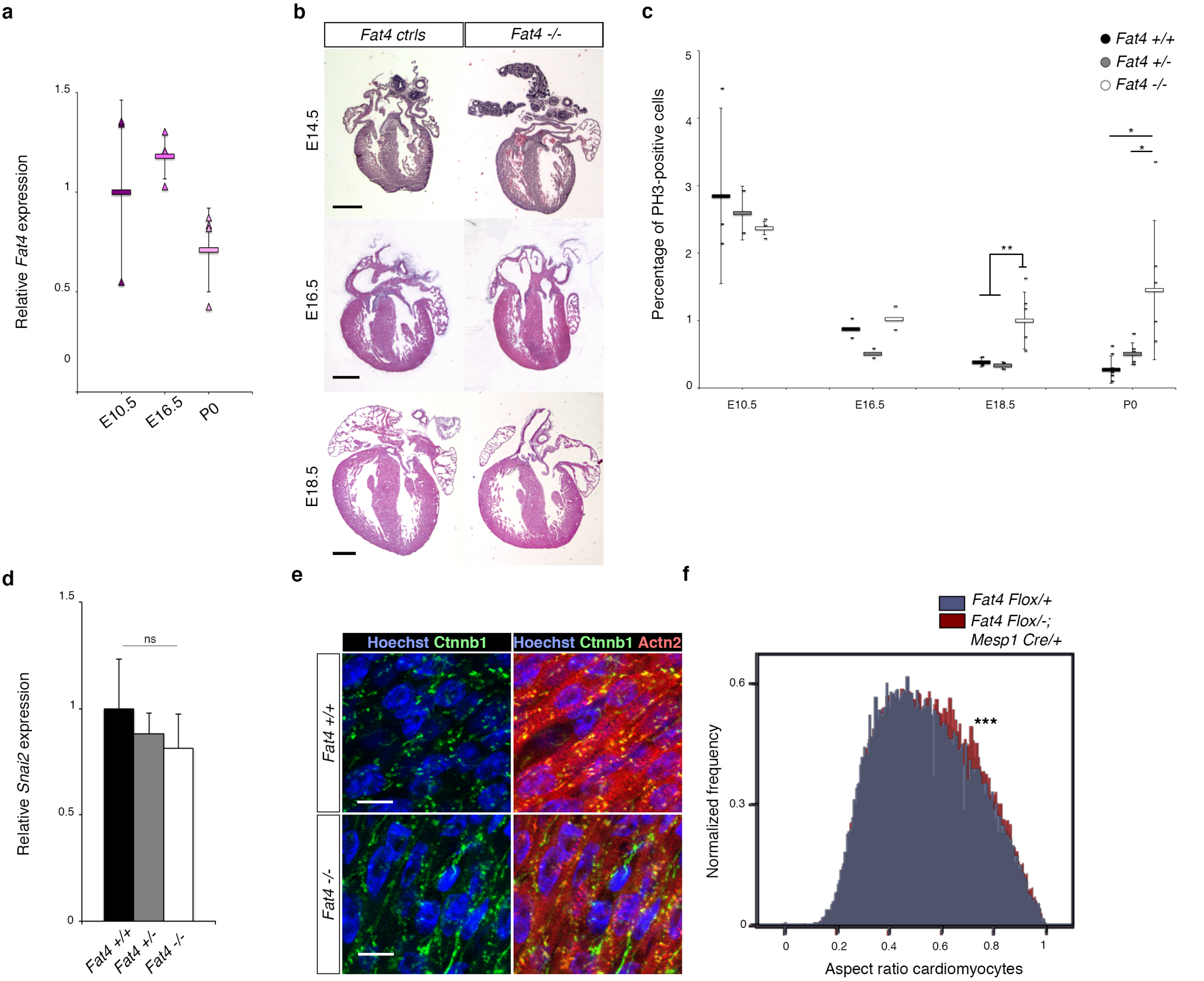


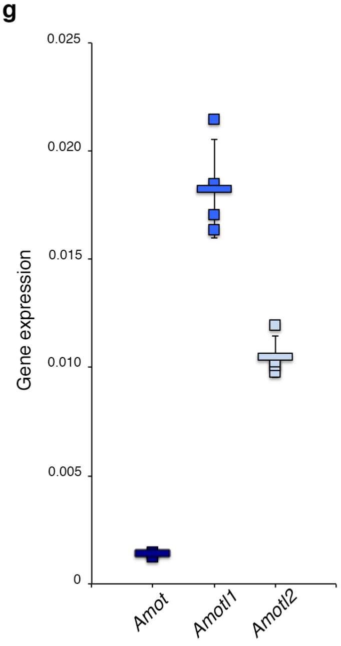


**Supplementary Figure 4. *Fat4* and *Amotl1* expression, kinetics of the phenotype of *Fat4* mutant hearts and normal activity of the Wnt pathway.** (a) Expression of *Fat4* in wild-type hearts at E10.5 (n=3), E16.5 (n=4) and P0 (n=4). Data are normalised to the level in E10.5 hearts. (b) Histological sections of control and mutant hearts at E14.5, E16.5 and E18.5. (c) Quantification of phosphorylated histone H3 (PH3)-positive cells at E10.5 (n=3 $Fat4^{+/+}$, 2 $Fat4^{+/-}$, 3 $Fat4^{-/-}$), E16.5 (n=2, 2, 2), E18.5 (n=3, 3, 5) and P0 (n=6, 6, 5). * $p<0.05$, ** $p<0.01$ (Anova). (d) Relative expression of the β-catenin target gene *Snai2* in $Fat4^{+/+}$ (n=5), $Fat4^{+/-}$ (n=5) and $Fat4^{-/-}$ (n=7) hearts at P0. ns, no significant difference (Student test). (e) Immunodetection of active β-catenin (Ctnnb1) in $Fat4^{+/+}$ and $Fat4^{-/-}$ hearts at P0. (f) Quantification of the aspect ratio between the minor and major axes of cardiomyocytes indicates rounder cells in mutant $Fat4^{Flox/-};Mesp1^{Cre/+}$ (n=112,792 cells pooled from 6 hearts) compared to control $Fat4^{Flox/+}$ (n=147,843 cells pooled from 7 hearts) samples at P14. *** $p<0.001$ (Z test). (g) Relative transcript levels of genes of the angiomotin family in wild-type hearts (n= 4) at E16.5. Scale bars : 500 $\mu$m in (b), 10 $\mu$m in (e).

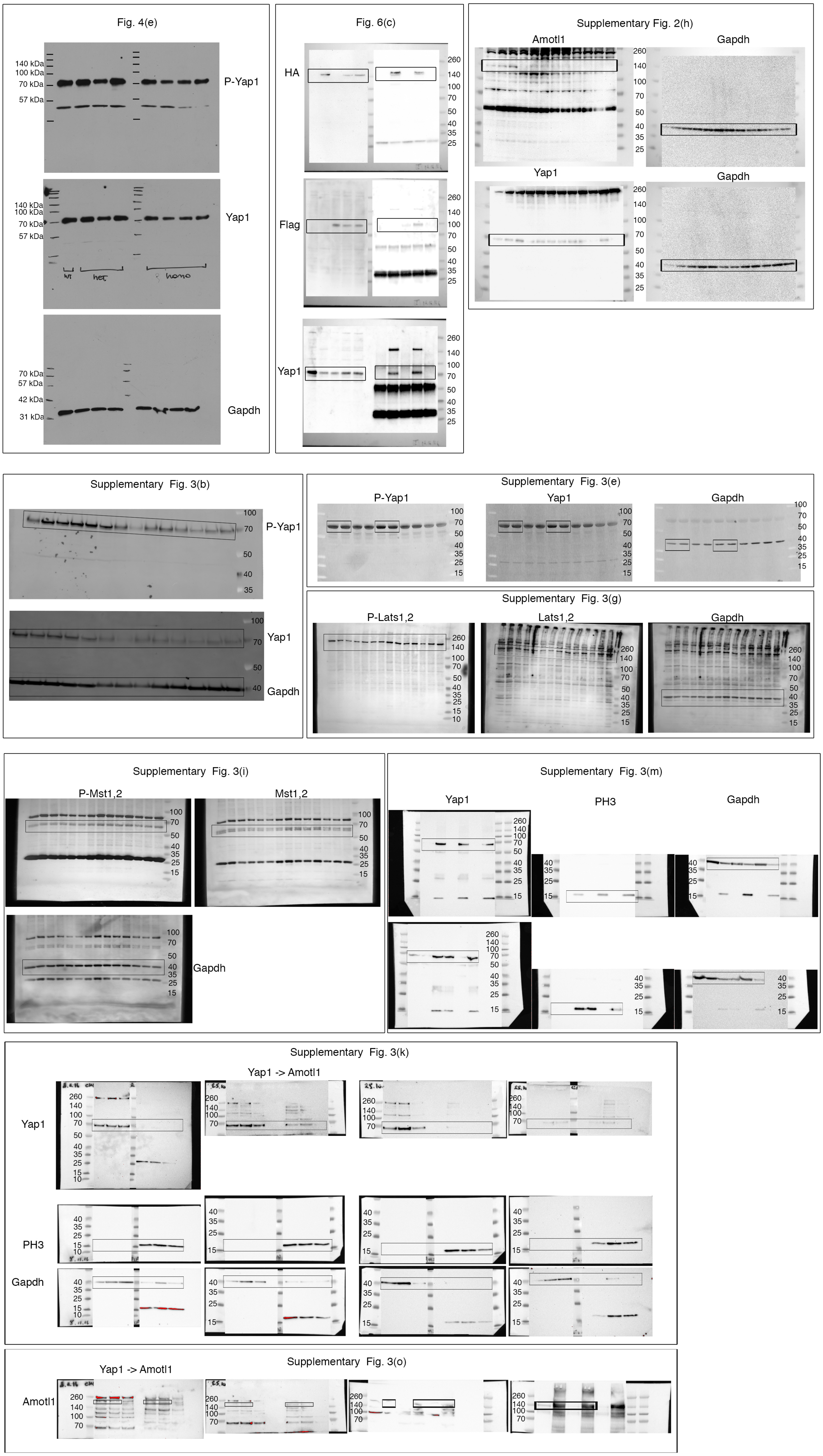


**Supplementary Figure 5. Original un-cropped Western blots are shown.**

**Supplementary Table 1. List of primer sequences used in RT-qPCR.**

| Species | Gene | Forward Primer | Reverse Primer |
|---|---|---|---|
| Mouse | Acta1 | GCATGCAGAAGGAGATCACA | ACATCTGCTGGAAGGTGGAC |
| Mouse | Amot | ACTGAGGGTCCTGCAAATCC | CAGGGGCATCTGGCTTATCT |
| Mouse | Amotl1 | CCCGCCTACTTCTACCCAGA | GGGTCCTCTACGCTTTTCCC |
| Mouse | Amotl2 | AAACTGCTTGCCCAGAGCTA | TCCAGCAGTTCAGCATGTCG |
| Mouse | Aurkb | GATTGCAGACTTTGGCTGGTC | ATTTCATTATGCATGCGCCCC |
| Mouse | Birc2 | TGAGAACTACAGGACCGTCAAT | TCTTCCGAATCAGTGATAAGTCA |
| Mouse | Birc5 | GAACCCGATGACAACCCGAT | TGGCTCTCTGTCTGTCCAGT |
| Mouse | Ccna2 | CCCGGAGCAAGAAAACCACT | TCATTAACGTTCACTGGCTTGT |
| Mouse | Cdc20 | CGCATTTGGAACGTCTGCTC | GCAAAGCCGTGACCTGAGAT |
| Mouse | Cdkn1b | GTTTCAGACGGTTCCCCGAA | CTTAATTCGGAGCTGTTTACGTCT |
| Mouse | CTGF | AGAACTGTGTACGGAGCGTG | GTGCACCATCTTTGGCAGTG |
| Mouse | Cyr61 | TTGACCAGACTGGCGCTCTC | AGCGCAGACCTTACAGCAG |
| Mouse | Fat4 | AGGACTTTGGTGGCATTGAG | GGGTCTGTTTTGGAGATGGA |
| Mouse | Gapdh | ACCCAGAAGACTGTGGATGG | CACATTGGGGGTAGGAACAC |
| Mouse | Myh6 | CAAGCTGCAGACAGAGAACG | TGCTGGGTGTAGGAGAGCTT |
| Mouse | NppA | ATCTGCCCTCTTGAAAAGCA | GCTCCAATCCTGTCAATCCT |
| Mouse | Nppb | TCGGATCCGTCAGTCGTTTG | TTCAAAGGTGGTCCCAGAGC |
| Mouse | Snai2 | ACTGGACACACACACAGTTAT | TGCCGACGATGTCCATACAG |
| Rat | Amotl1 | ACAAAGCTGCAGAGAGCCAT | TGCCAGCTCCATTTCCAACT |
| Rat | Fat4 | GGGGACAGATGTCCTGTTGG | TGAACTGTGAGTTTCCACCGA |
| Rat | Gapdh | AAGTTCAACGGCACAGTCAAG | TACTCAGCACCAGCATCACC |
| Rat | Yap1 | CTGCCCGACTCCTTCTTCAA | TGGAGACGAGTGAGCTCGAA |

| **Supplementary Table 2. Statistics reporting** | | | | | |
|---|---|---|---|---|---|
| Figure Panel or page | Test | S.D. or S.E.M. | n value (This number represents the sample size used to derive statistics. Please explain how samples were defined - cells, dishes, extracts) | Nb of times experiment was replicated in laboratory | p value |
| 1c | Anova | S.D. | Fat4+/+ n=7; Fat4 +/- n=5; Fat4-/- n=6 hearts | 2 | yes |
| 2b | Anova | S.D. | Fat4 +/+ n=6; Fat4 +/- n=6; Fat4 -/- n=5 hearts; more than 1500 cells per embryo were assessed | 4 experiments; 3 regions; pooled | yes |
| 2c | Student | S.D. | Ctrl n=7; mutant n=6 hearts | 1 | yes |
| 2d | Anova | S.D. | Fat4 +/+ n=5; Fat4 +/- n=5; Fat4 -/- n=7 heart RNA extracts | 1 | yes |
| 2e | Student | S.D | n=20 cultures (dishes) of Crtl and *Fat4* siRNA each, more than 900 cells per culture were assessed | 4 experiments; pooled | yes |
| 2g | Student | S.D. | Fat4 -/- n=6 ; Fat4 ctrl n=9 hearts. More than 40 cells per embryo were assessed | 3 | yes |
| 2h | Anova | S.D. | Fat4 +/+ n=5; Fat4 +/- n=5; Fat4 -/- n=7 heart RNA extracts | 1 | yes |
| 3a | Anova | S.D. | Fat4 +/+ n=5; Fat4 +/- n=5; Fat4 -/- n=7 heart RNA extracts | 2 | yes |
| 3d | Student | S.D | Crtl siRNA n=6; *Fat4* siRNA n=6 cultures. More than 11 cells per culture were assessed | 1 | yes |
| 3f | Student | S.D | n=9 and 14 cells transfected with pCIG and pFat4-2DECD-flag respectively | 2 experiments; pooled | yes |
| 3g | Anova | S.D | n=20, 20, 8 and 8 cultures (dishes) of Crtl, *Fat4, Yap1* and *Fat4+Yap1* siRNA respectively, more than 900 cells per culture were assessed. | Data were pooled from 4 independent experiments for Crtl and *Fat4* siRNA, and 1 experiment for *Yap1* and *Fat4+Yap1* siRNA. These experiments were done by pooling 3 different Yap1 siRNAs. Results with Yap1 siRNA-1 alone were similar (n=8; 2 experiments; not shown). | yes |
| 4b | Anova | SD | Ctrl n=6; mutant n=6; rescued n=6 hearts | 1 | yes |
| 4c | Anova | SD | Ctrl n=5; mutant n=5; rescued n=4 hearts | 1 | yes |
| 4d | Anova | SD | Ctrl n=7; mutant n=6; rescued n=3 hearts | 1 | yes |
| 4f | Anova | S.D. | Fat4 +/+ n=4; Fat4 +/- n=6; Fat4 -/- n=4 heart protein extracts | 2 | yes |
| 4h | Student | SD | trl siRNA n=6; Fat4 siRNA n=6 cultures (dishes). More than 3 transfected cells per culture were assesse | 1 | yes |
| 5e | Anova | S.D. | Fat4 +/+ n=3; Fat4 +/- n=3; Fat4 -/- n=3 hearts. More than 100 cells per heart were assessed. | 2 experiments; pooled | yes |
| 5f | Anova | S.D | n=20, 20, 16 and 16 cultures (dishes) of Crtl, *Fat4* , *Amotl1* and *Fat4+Amotl1* siRNA respectively, more than 900 cells per culture were assessed | Data were pooled from 4 independent experiments for Crtl and *Fat4* siRNA, and 3 for *Amotl1* and *Fat4+Amotl1* siRNA. | yes |
| 6b | Anova | SD | Ad-GFP+Ctrl siRNA n=8; Ad-nls Amotl1+Ctrl siRNA n=8; Ad-nls Amotl1+Yap1 siRNA n=8 cultures (dishes) | 1 | yes |

| Figure Panel or page | Test | S.D. or S.E.M. | n value (This number represents the sample size used to derive statistics. Please explain how samples were defined - cells, dishes, extracts) | Nb of times experiment was replicated in laboratory | p value |
|---|---|---|---|---|---|
| S1b | Wilcoxon U test | NA | 335 divisions (4 embryos), 524 divisions (3 embryos) | 1 | yes |
| S1c | NA | NA | 335 divisions (4 embryos), 524 divisions (3 embryos) | 1 | NA |
| S2a | Anova | SD | Fat4 +/+ n=6; Fat4 +/- n=6; Fat4 -/- n=5 hearts; | 4 experiments; 3 regions; pooled | yes |
| S2c | Student | SD | Ctrl n=6; mutant n=6 hearts | 1 | yes |
| S2e | Student | SD | Ctrl n=4; mutant n=4 hearts | 3 litters | yes |
| S2f | Student | SD | Ctrls n=5; mutant n=5 hearts | 2 | yes |
| S2g | Anova | S.D | For *Fat4* expression, n=6, 4,6 and 6 cultures (dishes) of Crtl, *Fat4* siRNA-1, *Fat4* siRNA-2 and Fat4 siRNA-3 respectively. For *Yap1* expression, n= 6, 6, 5, 6 cultures of Crtl, Yap1 siRNA-1, Yap1 siRNA-2 and Yap1 siRNA-3 respectively. For *Amotl1* expression, n=5, 6, 6, 4 cultures of Crtl and *Amotl1* siRNA-1, Amotl1 siRNA2 and Amotl1-siRNA3 respectively. | 4 experiments for Crtl and Fat4 siRNA-1, 3 experiments for Yap1 siRNA-1 and 2 experiments for all other conditions. | yes |
| S2i | Anova | SD | n=3 cultures for each condition | 1 | yes |
| S2k | Anova | SD | Ctrl siRNA n=20; *Fat4* siRNA-1 n=20; all others n=4 cultures | 4 experiments for Crtl and Fat4 siRNA-1, 1 experiment for all other conditions. | yes |
| S2m | Student | SD | Ctrl siRNA n=6; Fat4 siRNA n=6 cultures. More than 80 cells per culture were assessed. | 2 | yes |
| S3c | Anova | S.D. | Fat4 +/+ n=4; Fat4 +/- n=6; Fat4 -/- n=4 heart protein extracts | 2 | yes |
| S3d | Anova | S.D. | Fat4 +/+ n=5; Fat4 +/- n=5; Fat4 -/- n=7 heart RNA extracts | 1 | yes |
| S3f | Student | S.D | n=7 cultures (dishes) for each condition | 3 experiments; pooled | yes |
| S3h | Student | S.D. | Fat4 +/- n=7; Fat4 -/- n=7 heart protein extracts | 2 | yes |
| S3j | Student | S.D. | Fat4 +/- n=7; Fat4 -/- n=7 heart protein extracts | 1 | yes |
| S3l | Anova | S.D. | n=3 cultures (dishes) for each condition | 1 | yes |
| S3n | Student | S.D. | n=3 cultures (dishes) for each condition | 1 | yes |
| S4a | N/A | S.D. | E10.5 n=3; E16.5 n=4; P0 n=4 heart RNA extracts | 1 | N/A |
| S4c | Anova | S.D. | Fat4 +/+ n=3; Fat4 +/- n=2; Fat4 -/- n=3 E10.5 hearts | 2 experiments; 1 region; pooled | yes |
| | Anova | S.D. | Fat4 +/+ n=2; Fat4 +/- n=2; Fat4 -/- n=2 E16.5 hearts | 1 experiment; 2 regions; pooled | yes |
| | Anova | S.D. | Fat4 +/+ n=3; Fat4 +/- n=3; Fat4 -/- n=5 E18.5 hearts | 2 experiments; 2 regions; pooled | yes |
| | Anova | S.D. | Fat4 +/+ n=6; Fat4 +/- n=6; Fat4 -/- n=5 P0 hearts | 4 experiments; 3 regions; pooled | yes |
| S4d | Student | S.D. | Fat4 +/+ n=5; Fat4 +/- n=5; Fat4 -/- n=7 heart RNA extracts | 1 | yes |
| S4f | Z-test | N/A | Ctrls n=112,792 cells pooled from 6 hearts, mutants n=147,843 cells pooled from 7 hearts | 1 | yes |
| S4g | N/A | S.D. | wt E16.5 n=4 heart RNA extracts | 1 | N/A |
